# The Association Between Amygdala Subfield-Related Functional Connectivity and Stigma Reduction 12 Months After Social Contacts: A Functional Neuroimaging Study in a Subgroup of a Randomized Controlled Trial

**DOI:** 10.3389/fnhum.2020.00356

**Published:** 2020-08-27

**Authors:** Yuko Nakamura, Naohiro Okada, Shuntaro Ando, Kazusa Ohta, Yasutaka Ojio, Osamu Abe, Akira Kunimatsu, Sosei Yamaguchi, Kiyoto Kasai, Shinsuke Koike

**Affiliations:** ^1^University of Tokyo Center for Integrative Science of Human Behavior (CiSHuB), Tokyo, Japan; ^2^Center for Evolutionary Cognitive Sciences, Graduate School of Art and Sciences, The University of Tokyo, Tokyo, Japan; ^3^The International Research Center for Neurointelligence (WPI-IRCN), Institutes for Advanced Study (UTIAS), University of Tokyo, Tokyo, Japan; ^4^Department of Neuropsychiatry, Graduate School of Medicine, University of Tokyo, Tokyo, Japan; ^5^Department of Psychiatric Rehabilitation, National Center of Neurology and Psychiatry, National Institute of Mental Health, Kodaira, Japan; ^6^Department of Radiology, Graduate School of Medicine, University of Tokyo, Bunkyo City, Japan; ^7^Department of Radiology, IMSUT Hospital, The Institute of Medical Science, The University of Tokyo, Minato City, Japan; ^8^University of Tokyo Institute for Diversity and Adaptation of Human Mind (UTIDAHM), Meguro City, Japan

**Keywords:** amygdala, stigma, seed-based connectivity analysis, resting state functional MRI, randomized controlled trial

## Abstract

Social contact is one of the best methods for reducing stigma, and the effect may be associated with emotional response and social cognition. The amygdala is a key region of these functions and can be divided into three subregions, each of which has a different function and connectivity. We investigated whether the amygdala subregion-related functional connectivity is associated with the effect of anti-stigma interventions on reducing mental health-related stigma in a randomized controlled trial (RCT) over 12 months. Healthy young adults [*n* = 77, age, mean (*SD*) = 21.23 (0.94) years; male, *n* = 48], who were subsampled from an RCT (*n* = 259) investigating the effect of anti-stigma interventions, using filmed social contacts (FSC) or internet self-learning (INS), on reducing stigma, underwent 10 min resting-state functional magnetic resonance imaging between the trial registration and 12 months follow-up. The extent of stigma was assessed at the baseline, post-intervention and 12 month follow-up surveys, using the Japanese-language version of the Social Distance Scale (SDSJ), to assess negative emotional attitude toward people with schizophrenia. We compared associations between amygdala subregion-related functional connectivity and changes in the SDSJ scores for 12 months across the control, INS, and FSC groups. Associations between the change in stigma for 12 months and the superficial (SF) subregion of the amygdala-related connectivity in the intracalcarine cortex [(x, y, z) = (−8, −66, 12), z = 4.21, *P*_*FWE–corrected*_ = 0.0003, cluster size = 192] differed across groups. The *post hoc* analysis showed that the SF–intracalcarine cortex connectivity was negatively correlated with the change in stigma only in the FSC group. The current results indicate that greater SF–intracalcarine cortex connectivity is associated with a better response to the FSC interventions, suggesting that biological variability could underlie the long-term effect of anti-stigma interventions on stigma in the real world.

## Introduction

Stigma is defined as ignorance, stereotype, prejudice, and discrimination toward people in social minority groups, such as individuals of certain races or sexual orientation, or those with physical and mental health problems. Mental illness-related stigma is widespread globally and hinders the functional recovery of people with mental health problems, and also hampers early support and treatment of affected people seeking help (Thornicroft, 2003). Several randomised controlled trials (RCTs) have shown that contact-based intervention, i.e., to see a person with mental illness, is one of the best methods for reducing stigma (Thornicroft et al., 2016; Koike et al., 2018b). Theoretically, the stereotype content model can explain the effect of social contacts on reducing stigma (Fiske, 2012; Thornicroft et al., 2016; Koike et al., 2018b; Maunder and White, 2019). By social contact interventions, people without mental illness (intergroup) can know people with mental illness (outgroup), and the psychological gap between intergroup and outgroup could decrease through a sharing of common goals and values.

Previous studies have conceptualized the consequence of stigma that problem of knowledge may develop into problem of attitude and behavior (Thornicroft, 2003; Thornicroft et al., 2016). Social contacts have been thought to reduce stigma under this consequence. However, a 12 month follow-up study exploring the course of changes in stigma showed that, after social contact interventions, a decrease in negative emotional attitude toward people with schizophrenia was followed by an increase in practically useful knowledge and a decrease in the negative stereotype for the disease (Koike et al., 2018a). The results suggest that social contacts could first alter the emotional response toward people with mental illness, and then has a long-term effect on reducing stigma.

Brain researches using functional magnetic resonance imaging (fMRI) and event-related potentials have shown that activity in the amygdala (Quadflieg and Macrae, 2011; Forbes et al., 2012), insula (Krendl, 2016; Hughes et al., 2019), medial frontal cortex (Harris and Fiske, 2006), dorsolateral prefrontal cortex (Quadflieg and Macrae, 2011; Forbes et al., 2012; Hughes et al., 2019) and orbitofrontal cortex (Forbes et al., 2012) increase during the judgement of outgroup people as compared to that of intergroup people. These regions are closely linked to emotional responses and social cognitive processes (Bickart et al., 2011; Koban and Pourtois, 2014; Takano et al., 2017). Among these brain regions, the amygdala is a key brain region of bottom-up emotional response in which emotional stimuli arise (Balderston et al., 2015; Janak and Tye, 2015) and also plays a role in emotional decision-making (Seymour and Dolan, 2008), social cognition (Bickart et al., 2014) and complicated belief-changes or maintenance, such as maintenance of political beliefs (Kaplan et al., 2016). Therefore, the effect of social contact intervention in the real world may be affected by the amygdala.

**FIGURE 1:**
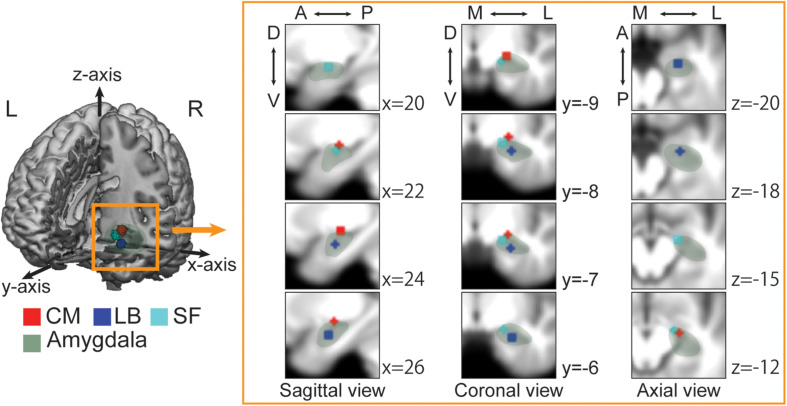
Each amygdala subregion imposed on the averaged anatomical image across all participants. The 3D image is cut out at the *x*-axis = 0, *y*-axis = 0, and *z*-axis = −33. The sagittal, coronal, and axial views depict regions of interest (ROIs) at the nearest coordinate of the right center of each ROI. A, anterior; P, posterior; D, dorsal; V, ventral; M, medial; L, lateral. Green, Whole amygdala; Red, Centro-medial (CM); Blue, Latero-basal (LB); Cyan, Superficial (SF). The laterobasal (LB) subregion is anatomically located in the lateral part of the amygdala and lies adjacent to the amygdalohippocampal area. The centromedial (CM) subregion is located in the dorsal and medial part of the amygdala and bordered dorsally by the globus pallidum and medially by the stria terminalis and the optic tract. The superficial (SF) subregion, known as cortical-like region, is located in the rostral part of the amygdala and connected to the lateral olfactory tract.

Recent studies have revealed that various amygdala functions are partially localized and explained by three subregions: the superficial (SF), latero-basal (LB) and centro-medial (CM) subregions (Figure 1; Sah et al., 2003). Each subregion is closely connected to the others and all subregions are associated with observing visual stimuli and discriminating faces (Bzdok et al., 2013), but are connected to different brain areas and contribute to different functions (Balderston et al., 2015; Janak and Tye, 2015). The SF subregion is mainly connected to the mesolimbic regions, ventromedial prefrontal cortex, medial temporal lobe, striatum and hypothalamus, and is thought to be related to the social affiliation network that is involved in prosocial sentiments (e.g., compassion or empathy) and in promoting fair social interactions (Bickart et al., 2012, 2014; Jiang et al., 2019). The CM subregion is connected to the anterior cingulate cortex, insular cortex, striatum, hypothalamus, and brain stem and is associated with the aversive network, which is implicated in avoidance behaviors (Bickart et al., 2012, 2014; Jiang et al., 2019). The LB subregion is widely connected to cortical and sub-cortical regions, including sensory regions, the orbital prefrontal cortex, hippocampus, and striatum and is related to the perception network (Bickart et al., 2012; Janak and Tye, 2015; Jiang et al., 2019). Given the role of the amygdala in emotional responses and social cognitive processes, each subregion could uniquely contribute to mental illness-related stigma; however, little has been examined the relationship between brain activity and the effect of interventions on reducing stigma in the real world.

We have previously shown the effect of filmed social contact interventions on reducing mental illness-related stigma over a 24 month period in an RCT (Koike et al., 2018b; Yamaguchi et al., 2019). In the secondary analysis, the effect may arise from changing emotional attitudes toward people with schizophrenia, followed by other components of stigma (Koike et al., 2018a), suggesting that amygdala-related functions may contribute to the change in stigma. However, to the best of our knowledge, the biological aspects of reducing stigma during psychosocial interventions have not yet been investigated with a long-term follow-up.

In this study, we combined a functional neuroimaging with the RCT of filmed social contact interventions. We tested the association between amygdala subregion-related functional connectivity and the effect of anti-stigma interventions in the RCT over 12 months. We hypothesized that the functional connectivity of each subregion would be uniquely associated with individual differences in changes in stigma after anti-stigma interventions.

## Materials and Methods

### Participants

**FIGURE 2:**
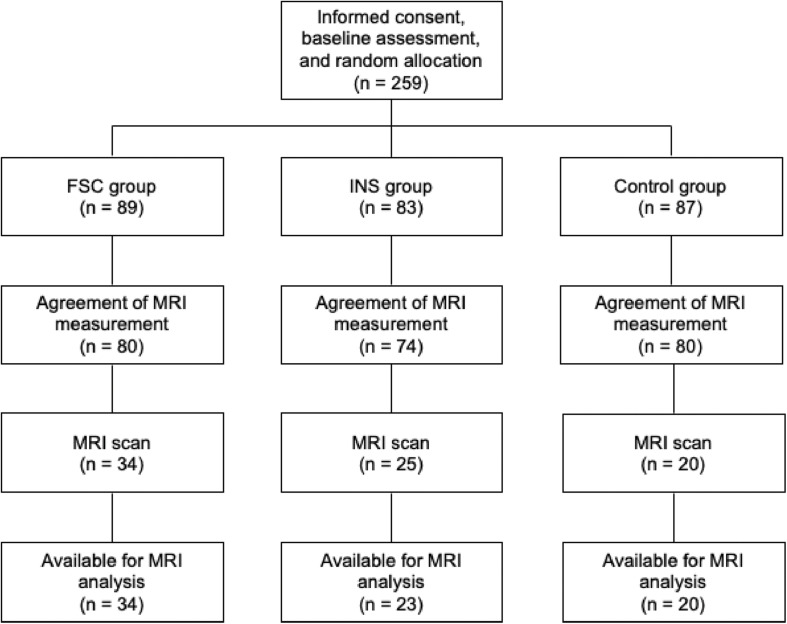
Participant flow in this study.

This study is a subsample measurement of brain imaging from a parallel-group RCT that examined the effect of social contact interventions on reducing mental illness-related stigma (Koike et al., 2018b; Yamaguchi et al., 2019) (UMIN-CTR Trial Number: UMIN000012239). Originally, 259 participants were registered and assigned to one of three groups [Control, Internet self-learning (INS), filmed-social contact (FSC); Figure 2 and Supplementary Table 1]. After the registration, all participants were asked whether they would be willing to participate in additional MRI experiments during the follow-up period, and 234 participants agreed to participate in these experiments. Of these, 79 participants underwent a 10 min resting-state fMRI (rs-fMRI) session between the post-intervention and 12 month follow-up surveys at the University of Tokyo Hospital. We excluded two participants from the INS group due to incomplete preprocessing fMRI data; thus, data from 77 participants were analyzed in this study [age at MRI measurement: mean (*SD*) = 21.23 (0.94) years; male, *n* = 48; Control, *n* = 20; INS, *n* = 23; FSC, *n* = 34, Table 1]. There was no difference in demographic characteristics and allocation between those who were originally registered in the RCT and those who were analyzed in this study (*p* > 0.05). Ethical approval was obtained from the Ethics Committee of the University of Tokyo (No. 15-116) and all participants provided written informed consent after receiving a full explanation of the study before RCT registration. Before the fMRI scan, all participants, and their parents, if the participants were aged less than 20 years, provided written informed consent under approval of the Faculty of Medicine, University of Tokyo [No. 3150-(28)].

**TABLE 1:** Demographic characteristics in this study.

	**Control group**	**INS group**	**FSC group**	***p*-value**
***N***	**20**	**23**	**34**	
Age at rs-fMRI scan [mean (*SD*), years]	20.94 (0.86)	21.36 (0.69)	21.31 (1.11)	0.274
Sex [female (%)]	7 (35.0)	9 (39.1)	13 (38.2)	0.958
Handedness [*n* (%)]				0.88
Right	12 (60.0)	17 (73.9)	22 (64.7)	
Mixed	6 (30.0)	4 (17.4)	8 (23.5)	
Left	2 (10.0)	2 (8.7)	4 (11.8)	
Sleepiness during the rs-fMRI scan [mean (*SD*)]	3.90 (1.21)	3.43 (1.16)	3.00 (1.03)	0.021
**The SDSJ score [mean (*SD*)]**				
Baseline	6.50 (3.19)	6.91 (3.07)	5.97 (2.85)	0.504
Post-intervention	6.75 (3.21)	5.61 (3.31)	3.91 (2.87)	0.005
12 months follow-up	6.24 (3.40)	5.05 (3.50)	4.31 (3.40)	0.182

**rs-fMRI, resting state functional magnetic resonance imaging; SDSJ, Japanese-language version of the Social Distance Scale; INS, Internet self-learning; FSC, filmed social contacts; SD, standard deviation.**

### Stigma Assessment

The extent of stigma was assessed at the baseline, post-intervention and 12 month follow-up surveys, using the Japanese-language version of the Social Distance Scale (SDSJ), to assess negative emotional attitude toward people with schizophrenia and focused on our previous study (Koike et al., 2018a). The SDSJ comprises five items rated on a 4-point Likert scale (range 0–15; higher scores represent a stronger desire for distance from people with schizophrenia, such as “It is best not to associate with a person with schizophrenia who has been in a mental hospital”) (Makita, 2006). All surveys were assessed using anonymous, self-administered questionnaires. Immediate change was calculated from post-intervention to baseline surveys, and long-term change from the 12 month follow-up to baseline. Therefore, a negative score for the change represents a greater effect of the intervention.

### Intervention

Following completion of the baseline survey, participants received individual laptop computers containing a 30 min intervention at a room in the University of Tokyo (Koike et al., 2018b; Yamaguchi et al., 2019). Briefly, the participant in the FSC group viewed a film including interviews with two men with schizophrenia, a portrayal of a woman with obsessive-compulsive disorder, general mental illness-related knowledge consistent with the interviews and portrayal and contact information for help-seeking. After the initial intervention, the participants received follow-up FSC interventions every 2 months (2, 4, 6, 8, and 10 months after registration) in the form of e-mails that contained a link to a website and medical information related to those illnesses.

The INS group was instructed to learn by searching for target terms (schizophrenia and mental illness) on the Internet, without any limit, using a laptop computer that were connected to the Internet. The participants also received follow-up e-mails at 2 month intervals, which instructed them to search for a specific keyword, determined according to the FSC interventions, on the Internet.

The control group played games on a laptop computer (e.g., mine-sweeper) and received no intervention during the follow-up period.

### MRI Acquisition

All MR images were obtained using a Discovery MR750w 3.0 Tesla scanner equipped with a 24-channel head coil (GE Healthcare, Waukesha, WI, United States). Each participant was instructed to focus on a fixation cross during the rs-fMRI scan. To obtain blood oxygen level-dependent (BOLD) fMRI data for the rs-fMRI scan, we applied the following pulse sequence: single-shot echo-planar sequence (TR/TE = 2500/30 ms, flip angle = 80°, field of view = 212 mm, matrix = 64 × 64, and voxel size = 3.3 × 3.3 × 4.0 mm^3^) with an acquisition time of 10 min 10 s. Forty contiguous slices were acquired in ascending order. The total number of recorded volumes was 244 for each participant. Immediately after the scan, each participant rated their sleepiness during the scan using a 7-point scale (1 = not at all, 7 = more than ever). After the rs-fMRI scan, each participant underwent a high-resolution anatomical MRI scan: T1-weighted 3D FSPGR (TR/TE = 7.7/3.1 ms, flip angle = 11°, field of view = 240 mm, matrix = 256 × 256 mm^2^, and voxel size = 1.0 × 1.0 × 1.2 mm^3^), with an acquisition time of 4 min 26 s.

### Image Preprocessing

#### Preprocessing for a Seed-Based Functional Connectivity Analysis

Conventional preprocessing was performed using tools from the FMRIB Software Library (FSL Version 6.0)^1^ package and the FieldMap Toolbox (Jezzard and Balaban, 1995; Hutton et al., 2002). fMRI data were preprocessed as follows: (1) distortion correction with fieldmaps, (2) head-motion correction by realigning the time series to the first volume, (3) removing non-brain tissues using the brain extraction tool, (4) slice-timing correction using Fourier-space phase shifting, aligning to the middle slice, (5) image smoothing with a 5 mm full-width at half-maximum Gaussian kernel, (6) linear detrending, and (7) grand-mean intensity normalization using a single multiplicative factor.

To remove the effects of time-points that were corrupted by large motions (motion outliers) from the analysis, a confounder matrix was created using FSL’s toolbox (FSL Motion Outliers) at the participant-level analysis. First, the rate of change in the BOLD signal across the entire brain at each data frame was calculated by first differentiating the volumetric time series and then calculating the root mean squared signal change over the whole brain (Power et al., 2012). This measure indexed the rate of change in the BOLD signal across the entire brain in each frame of data. Then, the threshold for motion outliers was applied to censor the data. The data-driven threshold was the 75th percentile + (1.5 interquartile range). This confounder matrix also included a time series extracted from individual white matter and cerebrospinal fluid regions. Before group analyses, the high-resolution anatomical image was normalized to the MNI avg152 T1-weighted template (2 mm isotropic resolution) using a non-linear transformation with a 10 mm warp resolution, as implemented in FSL’s fMRI non-linear registration tool.

#### Seed-Based Functional Connectivity Maps

We adopted anatomical templates from the SPM Anatomy toolbox (Amunts et al., 2005). We created a 3 mm sphere centered on the subregion of each sphere, merged the right and left sphere, and then set regions-of-interest (ROIs) (Figure 1). The centers of the left and right side of each ROI were [−21.3, −10.1, −9.07] and [23.7, −9.5, −9.21] for the CM, [−23.1, −6.46, −19.6] and [25.8, −5.07, −19.5] for the LB, and [−15.8, −9.43, −13.8] and [18.1, −7.96, −13.5] for the SF subregions.

Because of poor registration to the standard space from the individual rs-fMRI space, two participants in the INS group were discarded from analysis, and the total sample size for fMRI analysis was thus 77. Detailed demographics of the participants for the seed-based rs-fMRI analysis are described in Table 1. Time-series extraction from each subregion of the amygdala was performed on non-smoothed preprocessed rs-fMRI data. To create an individual functional connectivity map, the extracted time-series from each subregion was included in a general linear model (GLM) using FSL’s fMRI Expert Analysis Tool (FEAT). This model also included a confounder matrix as a nuisance covariate. We created four GLM models. We first entered the time-series from each ROI into an individual GLM model to create a connectivity map related to each ROI. Additionally, since each time-series could be correlated to each other, we also entered three time-series into one GLM model to regress out inter-ROI interaction.

### Statistical Analysis

Individual connectivity maps created by a GLM including time-series from all subregions were entered into a group level analysis. One-sample *t*-tests were performed to see the characteristics of three subregion related functional connectivity maps and confirm the validity of the subregion ROIs. Subsequently, we tested whether amygdala subregion-related connectivity maps were associated with immediate and long-term changes in the SDSJ, using the main effect of stigma change and group as well as interactions between these factors. We included age and sex as potential confounders. Three participants in the control group, one participant in the INS group, and two participants in the FSC group did not have an SDSJ score at the 12 months follow-up, and we imputed the missing scores using mean scores from each group. For all functional connectivity analyses, we set the cluster-forming threshold as *z* > 3.1. Clusters were then formed, their *p*-values calculated, and those *p*-values that were above the cluster *p*-threshold [*p* < 0.05, familywise error rate (FWE)-corrected] were disregarded. For significant connectivity, we then performed *post hoc* analysis to assess whether the relationship between functional connectivity and stigma change differed across the three groups.

## Results

### The Effect of the Intervention on Stigma

Similar to the previous findings (Koike et al., 2018b), a generalized linear mixed model showed significant group × time interaction (Immediate change: INS vs. Control, B = −0.29, SE = 0.24, *p* = 0.044; FSC vs. Control, B = −0.79, SE = 0.23, *p* < 0.001; Long-term change: INS vs. Control, B = −0.55, SE = 0.26, *p* = 0.030; FSC vs. Control, B = −-0.51, SE = 0.24, *p* = 0.035), but there was no significant main effect of group and time (*p* > 0.05). The mean sleepiness score during rs-fMRI scans was 3.44 (*SD* = 1.32). Head-motion during rs-fMRI sessions was calculated [mean ± *SD* (mm): (x, y, z) = (0.45 ± 0.05, 0.88 ± 0.72, 1.07 ± 0.74)] and three participants (two from the control group and one from the INS group) showed head-motion greater than 1 voxel size (3.3 × 3.3 × 4.0 mm^3^).

### Confirmation of Amygdala Subregion-Related Functional Connectivity Maps

**FIGURE 3:**
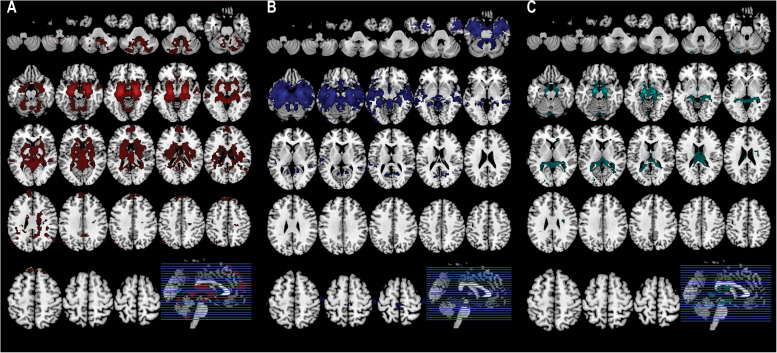
Each subregion-related connectivity map. **(A)** The CM-related connectivity, **(B)** the LB-related connectivity, **(C)** the SF-related connectivity.

The CM-related connectivity map included the amygdala, frontal pole, superior temporal gyrus, lingual gyrus, lateral occipital cortex, and cerebellum (Figure 3A). The LB-related connectivity map included the hippocampus, parahippocampus, middle temporal gyrus, and temporal pole (Figure 3B). The SF-related connectivity map included the amygdala and cerebellum (Figure 3C).

### Relationships Between the Change in Stigma Scores and the Amygdala Subregion-Related Connectivity Maps

**FIGURE 4:**
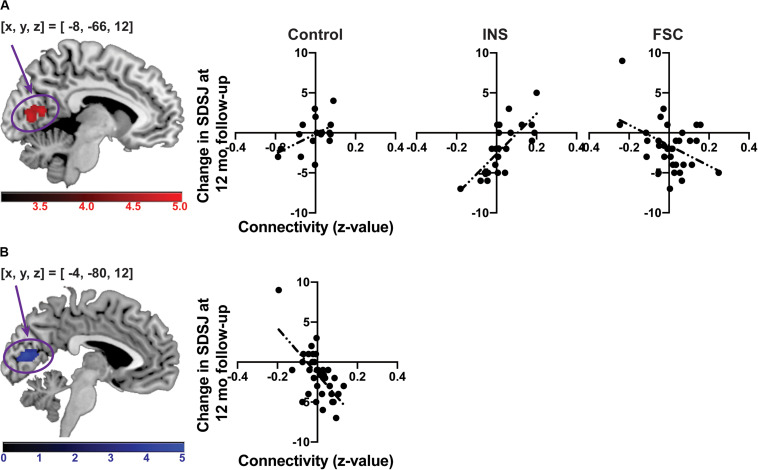
The associations between change in the SDSJ at 12 months follow-up and the SF-related connectivity. **(A)** The association between change in the SDSJ at 12 months follow-up and the SF-related connectivity in each group. **(B)** The association between change in the SDSJ at 12 months follow-up and the SF-related connectivity in the FSC group. The color-bars depict *z*-value. (x, y, z) shows a coordinate of peak voxel.

The *F*-test showed a significant interaction of long-term stigma change with group for the SF-related connectivity map in the intracalcarine cortex [(x, y, z) = (-8, −66, 12), z = 4.21, *P*_*FWE*__–__*corrected*_ = 0.0003, cluster size = 192, Figure 4A], although there were no significant main effects of stigma change and group. After Bonferroni correction of the performed GLMs (the GLM including the time-series from the CM, LB, SF or all ROIs), the cluster in the intracalcarine cortex remained significant (Bonferroni-adjusted significance = *P*_*FWE*__–__*corrected*_ < 0.0125). To confirm that head-motion did not affect the results, we discarded three participants who showed head-motion greater than 1 voxel in size. Even after these three participants were discarded, the interaction remained significant in the intracalcarine cortex [(x, y, z) = (-10, −74, 18), z = 4.28, *P*_FWE__–__*corrected*_ = 0.0006, cluster size = 172]. No other significant main effect of immediate and long-term changes and group were observed for any connectivity map.

A *post hoc* test revealed that a significant difference was present between the INS and FSC groups for the correlation between long-term stigma change and SF–intracalcarine cortex connectivity. The FSC group showed a significant correlation between change in the SDSJ score from baseline to the 12 months follow-up and the SF-related connectivity in the intracalcarine cortex (Figure 4B). No correlation was shown in the INS group. Thus, the result of the *post hoc* test was driven by the FSC group.

## Discussion

The present study found that the effect of social contact intervention on reducing stigma after 12 months, in an RCT, differed according to the amygdala subregion-related functional connectivity. The SF–intracalcarine cortex connectivity was negatively associated with changes in the SDSJ score between baseline and the 12 months follow-up in the FSC group, suggesting that participants with greater connectivity presented a better response to the social contact intervention. The reliability of the subregion-related functional connectivity was confirmed by showing a different pattern of the connectivity maps in our samples. To the best of our knowledge, no previous study has shown the relationship between amygdala subregion-related functional connectivity and the effect of the anti-stigma interventions using a combination of an RCT with fMRI.

The intracalcarine cortex is located in the occipital lobe and forms a part of the primary visual cortex (Dougherty et al., 2003). The amygdala has anatomical and functional connectivity with sensory regions, including the visual cortex (Pessoa and Adolphs, 2010; Bzdok et al., 2013). Amygdala connectivity to the visual regions plays important roles in processing affective visual stimuli (Pessoa and Adolphs, 2010) and could be associated with social cognition. An rs-fMRI study has shown that connectivity between the SF and the intracalcarine cortex is reduced in children and young adults with autism spectrum disorder (Rausch et al., 2016). In addition, the intracalcarine cortex, in concert with the amygdala, is activated when interpreting emotional cues from faces (Brandenburg-Goddard et al., 2014). Taken together, by combining socially relevant information, the SF–intracalcarine cortex connectivity seems to regulate the emotional reaction to visual stimuli.

The SF region contains the cortical nuclei and nucleus of the lateral olfactory tract, and is mainly involved in social affiliation (Bickart et al., 2012, 2014). The region evokes prosocial sentiments (e.g., compassion or empathy) to others and promotes prosocial behaviors, such as deciding to treat others fairly (Bickart et al., 2012, 2014). The SDSJ is designed to assess the extent of negative emotional attitude toward people with mental illness (Makita, 2006; Koike et al., 2018a). Therefore, the social affiliation toward people with mental illness could reduce the SDSJ scores. Contact-based interventions could decrease the stigma by decreasing the gap between the intergroup and outgroup (Fiske, 2012; Thornicroft et al., 2016; Koike et al., 2018b; Maunder and White, 2019). Taken together, due to the contact-based interventions, stigma may be decreased more in people with greater SF–intracalcarine connectivity, by improved understanding and evoking of prosocial sentiments toward people with mental illness.

Although amygdala-related function may focus on immediate emotional responses toward stimuli, no amygdala-related connectivity showed significant association with changes in the SDSJ at post-intervention. These results suggested that amygdala-related connectivity during the resting state may not reflect individual trait toward real-world emotionally evoked stimuli. In addition, amygdala–prefrontal cortex functional connectivity was not associated with any change in stigma in this study, suggesting that bottom-up emotional responses may be more related to stigma reduction by social contact interventions than top-down cognitive regulation. These results are in line with our previous follow-up study that these interventions first changed the negative emotional attitude toward people with schizophrenia, which then decreased stereotypes and increased useful information for the disease (Koike et al., 2018a).

This study had several limitations. First, since we did not acquire rs-fMRI data before and after the FSC intervention, we could not examine the causal relationship between the reduction in the SDSJ score and the amygdala-related neural network that provides a neurological underpinning to stigma. Further RCTs with planned MRI scans may elucidate the details of the neural substrate involved in formulating and decreasing stigma. Second, the sample size was not particularly large for within-group analysis. We calculated statistical power of correlation analysis between the connectivity values and SDSJ at follow-up in each group using G^∗^Power (Faul et al., 2007). *Post hoc* power analysis showed that statistical power was relatively large in all groups (0.8 in the control, 0.85 in the INS, and 0.95 in the FSC group). We thus assumed that statistical power in the current study was acceptable. In addition, for a task-based analysis, Desmond and colleague argued that about 12 subjects were required to achieve 80% power at the single-voxel level for typical activations (Desmond and Glover, 2002), while Thirion et al. showed that a minimum sample size of 20 is required to ensure acceptable reliability, while a sample size of 27 is preferred (Thirion et al., 2007). Given that a robust brain response or functional connectivity does not occur at rest, our sample size in the control and INS group was relatively small. Although a preferable sample size for rs-fMRI studies has not been addressed in the literature to date, an acceptable sample size may be more than 27 individuals per group, and future studies with a greater sample size are warranted.

To reduce mental illness-related stigma, the underlying biopsychological mechanism should be further investigated, as previous studies have mainly discussed the psychosocial context. Our findings suggest that biological variability could underlie the varied effects of anti-stigma interventions in the real world over the long-term. Addressing the biopsychological mechanism into psychoeducational curricula may strengthen the effect of anti-stigma campaigns in future.

## Data Availability Statement

All datasets presented in this study are included in the article/Supplementary Material.

## Ethics Statement

The studies involving human participants were reviewed and approved by the Faculty of Medicine, University of Tokyo. The patients/participants provided their written informed consent to participate in this study.

## Author Contributions

YN and SK designed the work, conducted statistical analyses, and wrote the draft manuscript. NO, KO, OA, AK, and KK contributed to MRI measurements. SA, KO, YO, SY, and SK contributed to conducting the RCT. All authors reviewed the draft manuscript critically and approved the final version of the manuscript.

## Conflict of Interest

The authors declare that the research was conducted in the absence of any commercial or financial relationships that could be construed as a potential conflict of interest.

## Footnotes

1 http://www.fmrib.ox.ac.uk/fsl/
