# Supplementary material for: The Association Between Amygdala Subfield-Related Functional Connectivity and Stigma Reduction 12 Months After Social Contacts: A Functional Neuroimaging Study in a Subgroup of a Randomized Controlled Trial
Source: Front Hum Neurosci. 2020 Aug 27;14:356. doi: 10.3389/fnhum.2020.00356 (PMC7481372; doi:10.3389/fnhum.2020.00356)
Supplement: Supplementary file 1 [file Data_Sheet_1.PDF]

Supplementary Table 1. **Demographic characteristics in this study and the original recruitment samples.**

	Initial recruitment	Rs-fMRI experiment	p-value
**N**	182	77	
**Age at the survey (mean [S.D.], years)**	19.94 (1.33)	20.12 (0.90)	0.290
**Sex (female [%])**	80 (44.0)	29 (37.7)	0.424
**The SDSJ scores at the baseline (mean [S.D.])**	5.89 (2.69)	6.39 (3.00)	0.188
**Allocation (%)**			0.078
**Control group**	67 (36.8)	20 (26.0)	
**INS group**	60 (33.0)	23 (29.9)	
**FSC group**	55 (30.2)	34 (44.2)	

SDSJ, Japanese-language version of the Social Distance Scale; INS, Internet self-learning; FSC, filmed social contacts; S.D, standard deviation.
